# Incorporating staffing instability in the nursing home Five-Star Staffing Composite

**DOI:** 10.1093/haschl/qxae159

**Published:** 2024-12-16

**Authors:** Dana B Mukamel, Debra Saliba, Heather Ladd, R Tamara Konetzka

**Affiliations:** iTEQC Research Program, Department of Medicine, Division of General Internal Medicine, University of California, Irvine, Irvine, CA 92617-3056, United States; Department of Medicine, UCLA Borun Center, David Geffen School of Medicine, University of california, Los Angeles, CA 90095-1687, United States; Veterans Administration Geriatric Research Education and Clinical Center (GRECC), Los Angeles, CA 90073, United States; RAND Health, Santa Monica, CA 90401, United States; iTEQC Research Program, Department of Medicine, Division of General Internal Medicine, University of California, Irvine, Irvine, CA 92617-3056, United States; Louis Block Professor of Public Health Sciences and the College, Department of Medicine, The University of Chicago, Chicago, IL 60637-1447, United States

**Keywords:** nursing homes, quality report cards, staffing

## Abstract

Staffing is an important indicator of nursing home quality and resident health outcomes. The Five-Star staffing ratings in Nursing Home Care Compare, the report card published by the Centers for Medicare and Medicaid Services, is based on average hours per resident-day and turnover measures. Studies have shown that a new measure of staffing instability, capturing day-to-day staffing variation, is associated with resident outcomes and provides additional information about quality not reflected in the current Five-Star staffing ratings. In this paper we simulate the impact of including the new staffing instability measure on staffing ratings for 13 641 nursing homes nationwide, using data for the third quarter of 2023. We found that, under a conservative scenario, 21% of nursing homes perform well or poorly enough on instability that this addition would change their current staffing rating, providing consumers with additional information about quality for these facilities, with minimal disruptions to the rating system as a whole. We also demonstrate that the choice of weights for each of the measures included in the Five-Star ratings matters. These weights should reflect policy priorities. We conclude that the Centers for Medicare and Medicaid Services can and should add staffing instability to its Five-Star staffing ratings.

## Introduction

The 1986 Institute of Medicine’s report on “Improving the Quality of Care in Nursing Home”^1^ shifted the quality culture in nursing homes from sole reliance on monitoring process and structure measures of quality,^2^ such as average staffing, to adding the importance of patient health outcomes and their relationship to processes of care and quality reporting. Over a decade later, in 1998, the Centers for Medicare and Medicaid Services (CMS) launched Nursing Home Compare,^3^ the forerunner of today's Nursing Home Care Compare (NHCC) and the flagship of all CMS’ series of the Compare report cards. The first report cards were still focused on health inspections and average staffing. But, by 2003, several outcome-based quality measures, such as rates of activities of daily living and pressure ulcers were added. By 2008, at the direction of Congress, and recognizing that too much information can make consumers’ decisions suboptimal,^4,5^ CMS introduced the Five-Star ratings. These ratings are a set of measures that summarize the individual quality measures into 4 measures^6^: an overall measure and 3 composites capturing 3 domains—staffing, resident outcomes, and health inspections.^7^ Since then, CMS regularly reviews the relationship of these measures to nursing homes’ performance, considers new data, and adjusts them as needed.

In July 2022, CMS introduced major enhancements to the Five-Star Staffing Composite, enabled by new Payroll-Based Journal (PBJ) data.^8^ The PBJ provides daily staffing hours per resident-day (HPRD) for all Medicare and Medicaid certified nursing homes. Prior to the PBJ, the staffing domain was based on the average staffing HPRD among registered nurses (RNs) and total nurse staffing, drawn from point-in-time data collected during annual inspections. These data have known limitations and biases.^9-12^ The PBJ provides more accurate staffing HPRD data and allows development of important new measures, including weekend staffing levels and staff turnover^13^ (eg, percentage of staff leaving the nursing home in 1 year). These measures have been researched extensively for decades and were shown to be associated with poor quality, capturing variations in staffing and affecting quality in ways not captured by annual staffing averages.^13-19^ Inclusion of these additional measures in the Five-Star Staffing Composite was one of the most significant changes that CMS has implemented in the rating system.

Yet, the measures currently included in the Staffing Composite are still limited. In particular, they are based on measures averaged over long periods of time (eg, 1 year). They capture the day-to-day experience of residents only in a limited way. The weekend staffing measure, while measuring the decline in staffing complement relative to weekdays, does not account for the possibility that staffing might also vary during weekdays.^12,20^ And variability during the week might actually be more detrimental to quality for several reasons. First, in many nursing homes the weekend lower staff complement is actually planned and there might be backup procedures in place if things go awry. Second, during the week there is “more happening” to residents, in terms of therapies, treatments, and activities, so less than a full staffing complement is more likely to lead to injuries or poor outcomes. Furthermore, there are more weekdays than weekend days. Thus, the exposure to low staffing days during the week compared with the weekend is likely to result in more poor outcomes than during the weekend.

Turnover, on the other hand, is a different type of disruption in care. It may or may not be associated with a reduction in staff, depending on whether or not the separation is initiated by the employee or the employer. If it is initiated by the employer or if the employer is given notice and can prepare by hiring or contracting for a replacement, there is likely no disruption in staffing levels. There is, of course, discontinuity in care by familiar staff, which has been of major concern with turnover's impact on quality.^19,21,22^ This is a particular issue for long-term residents and those with dementia who may exhibit anxiety and other behavioral issues if caregivers are turning over often and lack specialized training.

Recently, a new staffing measure, staffing instability,^20,23^ was developed to address this gap in capturing the daily variation in staffing, which is not addressed by either turnover or weekend staffing. In this paper we discuss the rationale for adding this measure to the Five-Star Staffing Composite and examine the implications of doing so.

### The case for expanding the Five-Star Staffing Composite

#### Defining staffing instability

Staffing instability, ie, the daily variation in staffing level in a nursing home, can be defined in different ways.^20^ The most intuitive definition is based on a count of the number of days in a period in which the facility's staff is below its average. Such a measure is also very easy to operationalize in a continuous quality-improvement program, by setting a staffing-level target and developing management operations designed to monitor and keep staff each day within a certain range of this target. In fact, such a measure is based on quality-control concepts used in industrial quality-improvement processes. It is designed to keep inputs into production on an even keel, reflecting the notion that best practices do not allow for wide, disruptive variations in inputs.^20^ In our prior studies we considered 2 alternative measures.^20,23^ The first is a measure that also includes days that exceed the target. This measure is less appropriate because, in many cases, the harm caused to residents on low staffing days cannot be mitigated by extra staff on the next day (eg, a fall leading to a broken hip due to a shortage of aides will not be reversed by more aides the following day).^20^ Another alternative is the coefficient of variation. This is a statistical construct, which averages the variation across the whole period (including high- and low-staffing days) rather than tying the staffing disruption to specific days. In addition to being less intuitive for nursing home management, it has been shown to have the lowest impact in terms of its average marginal effect on outcomes when compared with the 2 other measures.^20^

Based on these considerations we define staffing instability as “the percentage of days in a period (eg, quarter) in which staff HPRD are 20% or more below the facility average in the quarter.”

#### Are the staffing instability measures associated with resident health outcomes?

Improvement in the instability measures for certified nurse assistants (CNAs) and licensed practical nurses (LPNs) was shown to be associated with improved resident risk-adjusted health outcomes (while controlling for average HPRD of RNs, CNAs, and LPNs and other nursing home characteristics).^23^ Outcomes included pressure ulcers, worsening activities of daily living, hospitalization, and emergency department (ED) visits for long-stay residents, and antipsychotic drugs, failure to improve functioning by discharge, rehospitalizations, and ED visits for short-stay residents. Deficiency scores issued by state inspectors for quality problems also increased with instability.

#### Do the instability measures provide additional information about quality above and beyond the average staffing and turnover measures?

Mukamel et al^20^ used the average staffing measure and the instability measure to rank nursing homes into deciles from worse to best. Thus, each nursing home had 2 staffing rankings: one based on average staffing and one based on the instability measure. For example, a nursing home might have been ranked in the first quality decile by average staffing but on the fifth quality ranking by the instability measure. Agreement on the rankings for each nursing home was quantified by the weighted kappa. The weighted kappa can range from −1 to 1, with 0 indicating no agreement and 1 indicating perfect agreement.^20,24^ The weighted kappa measuring agreement between average staffing and instability for RNs was 0.513 (95% CI: 0.505–0.521), indicating poor to moderate agreement, and between average staffing and CNAs was 0.252 (95% CI: 0.243–0.260), indicating poor agreement.^25,26^ These values suggest that the instability measures and the average staffing measures have poor agreement on the ranking of nursing homes and, therefore, offer different perspectives about nursing homes’ quality and should both be included in the Five-Star Composite.

Sinha et al^27^ examined the same question with respect to turnover. They found a weak correlation between turnover and the instability measure. They estimated separate regression models predicting 6 long-stay and 4 short-stay quality measures as dependent variables. Total average staffing hours, total staffing turnover, and total staffing instability were the independent variables of interest, controlling for facility characteristics. They found that turnover and instability had independent associations with quality, with turnover more highly associated with some outcomes and instability more highly associated with other outcomes. Using our less preferred measure of instability, Brunt and Bowblis^13^ tested a similar research question and came to similar conclusions. This suggests that turnover and staffing instability capture different and non-overlapping aspects of quality and should both be included in the Five-Star Composite.

These studies suggest that the 3 measures—average staffing, turnover, and staffing instability—offer different and complementary perspectives on the impact of staffing on resident outcomes and nursing home quality. An evaluation that does not include all 3 dimensions might be incomplete. Decisions made by government regulators, health plans contracting services, and consumers selecting a nursing home will be partially misinformed, and to the degree that this attribute is important to these decision makers, biased in terms of the quality choices they believe they make. This suggests that the Five-Star Staffing Composite, intended to help users of the NHCC considering staffing information, should include all 3 components.

## Data and methods

This study re-creates the NHCC Five-Star Staffing Composite according to CMS specifications, adding an instability measure, and assesses the impact that this addition has on the ratings.

### CMS methodology for calculating the Five-Star Staffing Composite

The Five-Star Staffing Composite is made of 6 individual quality measures.^7^ Two are based on average nursing HPRD: RN and total nursing (RNs + LPNs + CNAs) HPRD. A third measure includes total nursing HPRD on weekends. The other 3 measure turnover: percentage of RNs that left the nursing home over a 12-month period, percentage of total nursing staff who left, and number of administrators who left.

To create the Five-Star Staffing Composite, each of the 6 measures is assigned points (weights) as follows: RN and total nursing HPRD can obtain between 10 to 100 points each. Weekend total nursing, total nursing turnover, and RN turnover can obtain 5–50 points each, and administrator turnover can obtain 10–30 points. Actual points for each measure for a specific nursing home depend on the facility's decile in the national distribution. For example, a nursing home in the 64th percentile in terms of its RN HPRD, is in the sixth decile and will, therefore, be allotted 60 points. Once the points have been determined for all individual measures for the nursing home, they are summed up to provide the total Five-Star points for this facility. A nursing home that scores the maximum points for all individual measures will obtain a score of 380.

**Figure 1.**
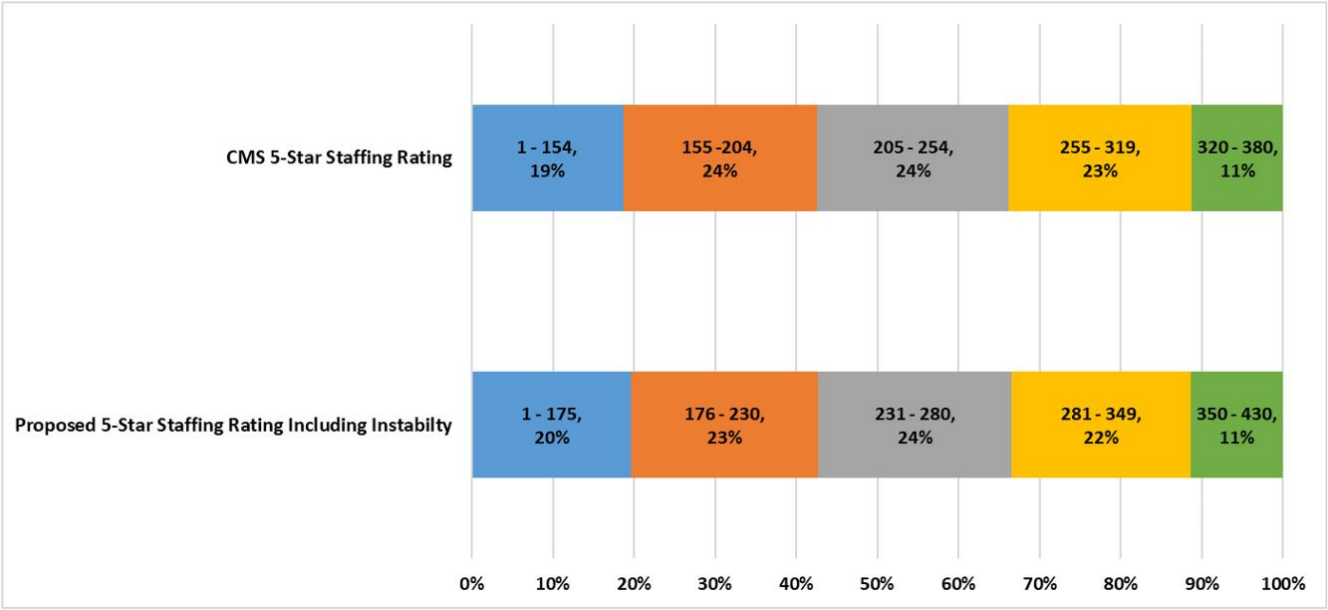
Distribution of nursing homes by star rating with associated point ranges. Source: Authors’ calculations. One star (in blue); 2 stars (in orange); 3 stars (in gray); 4 stars (in yellow); 5 stars (in green). Abbreviation: CMS, Centers for Medicare and Medicaid Services.

The points are then translated into Five-Star ratings, as shown in the top horizontal bar of Figure 1. This translation of points into stars is determined by CMS, reflecting its policy objectives and expectations of the distribution of nursing homes along the continuum of staffing from very low staffing level corresponding to “much below average” quality (ie, 1 star) to “much above average” staffing levels (ie, 5 stars).

### Simulation adding the instability measure to the Five-Star Staffing Composite

We used PBJ data for all 13 641 Medicare and Medicaid certified nursing homes without data errors in the third quarter of 2023 to re-create the CMS Staffing Composite (see Appendix A).

For simplicity, we calculated instability for total nurse staffing (RNs + LPNs + CNAs) as opposed to individual staffing types, consistent with prior papers,^27^ and calculated instability over the prior calendar quarter given that many NHCC measures are reported quarterly. Instability is defined as the percentage of days in the quarter in which total nurse staffing HPRD was 20% or more below the facility average total nurse staffing HPRD.^7^

The most consequential and subjective decisions relate to the number of points assigned to instability vs the other staffing measures, so we present several possibilities. We chose a base case assigning similar weight to instability as CMS assigns to most other recently added staffing measures, 5–50 points. We discuss 2 alternatives with weight decreased to 10–30 points or increased to 10–100 points, the 2 other weights used in the composite. Mechanically, we expect the former to have the least impact on the Five-Star Staffing Composite rating and the latter to have the most.

Following the CMS methodology, we used the national distribution of the instability measure to assign points to each nursing home. Nursing homes with the highest (worst) instability values in the 90th percentile received 5 points. Nursing homes with the lowest (best) instability values in the 0–10th percentile received 50 points.

To determine the new Five-Star Staffing Composite rating the instability points were added to the current CMS published score for each nursing home. The total possible number of points for the new Five-Star Composite is 430 compared with the current CMS Composite that has a maximum of 380 points. Star ratings for each nursing homes were assigned, mimicking the CMS distribution, as can be seen in the second bar of Figure 1. We note, however, that our assumption that CMS will choose the same distribution for the Five-Star Staffing Composite when adding the instability measure is ultimately a policy decision. If a different distribution were to be chosen, it would influence the results we report below.

### Analyses

We examined the impact of including instability in the Five-Star Staffing Composite by comparing the number of nursing homes that remain in the same Five-Star category, gain stars, or lose stars for the base case and 2 alternative cases. For the conservative base case at which the instability measure is weighted at 5–50 points and the less conservative case in which it is weighted at 10–100 points (as is the case for total RN average staffing measures), we also present a stratified analysis by nursing home characteristics.

## Results

Table 1 describes the sample characteristics. The majority (87%) had 50–249 beds, 68% were in the Midwest or South Census regions, 97% were free-standing, 72% were for-profit, and 59% were chain affiliated. Thirty-three percent had a 4- or 5-star Staffing Composite rating, 51% had a 4- or 5-star Quality Measures Composite, and 33% had a 4- or 5-star Health Inspections Composite ratings.

**Table 1.** Descriptive statistics: nursing homes characteristics.

	Number	Percentage
Bed size		
Less than 50	1452	11
50–99	5158	38
100–249	6685	49
250–499	319	2
500 or more	27	<1
Total	13 641	
Census region		
Northeast	2332	17
Midwest	4353	32
South	4879	36
West	2070	15
US Territory	7	<1
Total	13 641	
Chain		
Yes	7843	59
No	5538	41
Total	13 381	
Hospital-based		
Yes	451	3
No	13 190	97
Total	13 641	
Ownership		
For-profit	9818	72
Nonprofit	3003	22
Government	820	6
Total	13 641	
Quality Measures Composite ratings		
1 star	1042	8
2 stars	2069	15
3 stars	2967	22
4 stars	3562	26
5 stars	3911	29
Total	13 551	
Health Inspections Composite ratings		
1 star	2612	19
2 star	3349	25
3 star	3123	23
4 star	3146	23
5 star	1411	10
Total	13 641	

Source: Authors’ calculations.

We compared the current CMS Five-Star Staffing Composite rating with the new ratings that include the instability measure (see Table 2, base case). Overall agreement was high, at 79% of cells. The highest rate of agreement was at the extremes: 85% agreement on 1-star nursing homes and 82% agreement on 5-star nursing homes, possibly due to ceiling and floor effects. The lowest agreement, at 73%, was on the 3-star ratings. The percentage of nursing homes with any star ratings under the current formulation that would gain a star and the percentage that would lose a star was almost the same at 11.4% and 10.0% respectively. It is also noteworthy that no nursing home gained or lost more than 1 star.

**Table 2.** Comparison of the distribution of nursing homes by the current CMS Five-Star Staffing Composite rating with the base case (weight of 50 points) Five-Star Staffing Composite rating with instability.

	Five-Star Staffing Composite rating with instability					
Current CMS Five-Star Staffing Composite rating	1	2	3	4	5	Total
1	2278 (85%)	400 (15%)	0	0	0	2678
2	268 (9%)	2424 (77%)	453 (14%)	0	0	3145
3	0	439 (13%)	2378 (73%)	439 (13%)	0	3256
4	0	0	385 (13%)	2368 (79%)	260 (9%)	3013
5	0	0	0	276 (18%)	1273 (82%)	1549
Total	2546	3263	3216	3083	1533	13 641

Abbreviation: CMS, Centers for Medicare and Medicaid Services.

Row percentages in parentheses. Source: Authors’ calculations.

Results showed similar patterns in the 2 alternative cases (see Appendix B Tables 1 and 2) but the agreement percentages differed substantially. Using the maximum points for the instability measure as 30 rather than 50 as in the base case, we obtained higher overall agreement percentages at 86% compared with 79% in the base case, and a larger discrepancy between overall 1-star losers at 5% and 1-star gainers at almost twice as many—9%—compared with 11.4% and 10.0% in the base case. When the maximum points were 100 there was much less agreement overall at only 59%, with 19% of nursing homes losing 1 star and 22% gaining 1 star. In this case, a few (31; 0.2%) nursing homes, actually changed by 2 stars. These cases show that the weight is important and influences which nursing homes are “gainers” and “losers.”

**Figure 2.**
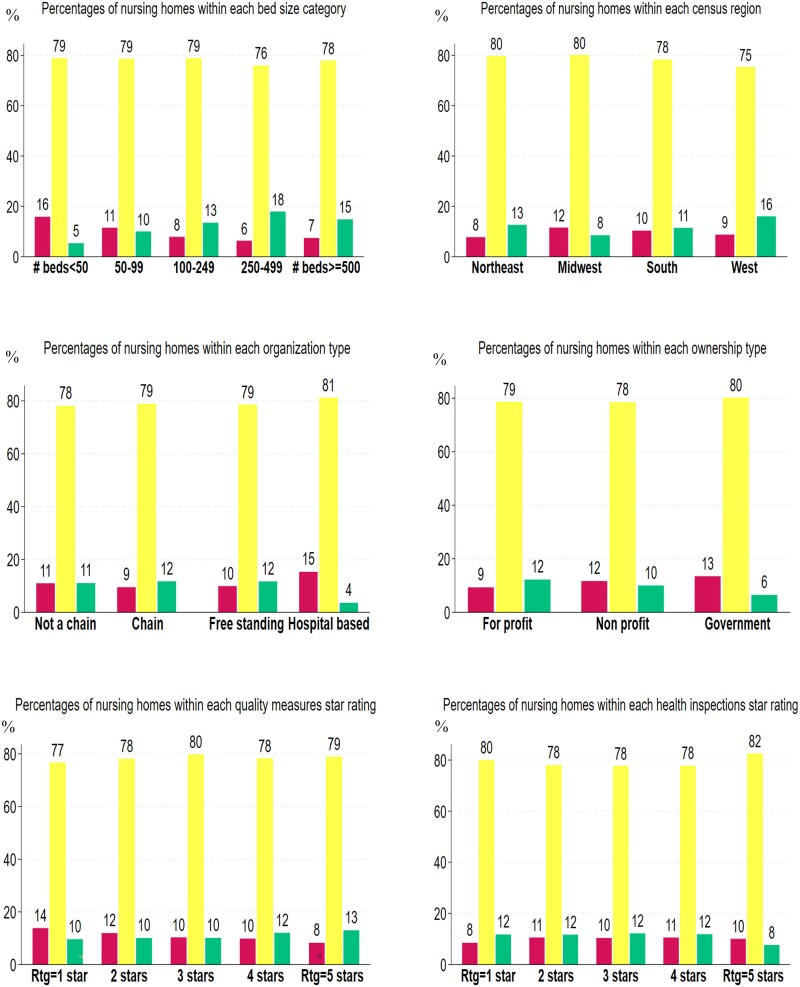
Impact of including instability in the Nursing Home Care Compare staffing composite by nursing home characteristics—simulations’ results. Source: Authors’ calculations. The figures were generated using statistical software. Nursing home will: Lose 1 star (in red); No change in stars (in yellow); Add 1 star (in green). Base case: Instability weight in the composite = 50 points. Abbreviation: Rtg, rating.

Figure 2 shows how adding the instability measure with a weight of 50 (base case) is distributed by nursing home characteristics. In green are the percentages of nursing homes within each category that gained 1 star, in yellow the percentages of those that retained the same number of stars, and in red those that lost 1 star. For example, in the chart showing the distribution within bed-size less than 50, 16% of nursing homes lost 1 star, 79% retained the same number of stars, and 5% gained 1 star. In general, we found that the vast majority of nursing homes, approximately 80%, were not affected by the addition of the instability measure, as suggested in Table 2. However, depending on facility characteristics, there were both winners and losers among the other 20%.

In terms of bed-size, we found that the small nursing homes, with fewer than 50 beds, had more losers (16%) than gainers (5%). The next category, 50–99 beds, was almost equal, with 11% losers and 10% winners. All other 3 categories had much larger percentages of winners, which is particularly large for those with 250–499 beds, with only 6% losing 1 star and 18% gaining a star.

In the Western part of the country and the Northeast we found substantially more gainers than losers (13% vs 8% and 16% vs 9%, respectively); in the South they were about equal (10% and 11%), but in the Midwest there were substantially more losers (12% vs 8%) than gainers.

Chain affiliation had a minimal association with gainers and losers. Nursing homes that were not part of a chain had 11% for both losers and gainers. Chain affiliation was associated with a 9% probability of losing a star and 12% of gain a star. Similarly, free-standing nursing homes had a similar percentage of losing a star at 10% and gaining a star at 12%. On the other hand, hospital-based nursing homes were much more likely to lose a star at 15% than to gain a star at 4%.

Ownership was also not associated with very large differences between losing and gaining percentages, except for government-owned facilities, which were much more likely to lose a star at 13% than to gain a star at 6%. For-profit facilities had losers at 9% and winners at 12% and nonprofits showed the opposite trend with 12% of losers and 10% of winners.

The Five-Star Quality Composite ratings showed a U-shaped trend, with more losers for the low-quality rating of 1 star at 14% compared with 10% winners, leveling off in the middle, and switching at the high-quality rating of 5 stars with 8% for losers and 13% for gainers. Interestingly, we found the opposite trend with respect to the Five-Star Health Inspections rating. This relationship was an inverted U-shape, with a lower loser percentage at a rating of 1 star of 8% and gainer percentage of 12%, leveling off in the middle, and having 10% losers in the high quality of 5 stars and only 8% of winners at the end of the Five-Star Health Inspections rating.

The Overall Five-Star (Appendixes C1 and C2) presents a pattern similar to the Five-Star Health Inspections, as the Overall and the Five-Star Health Inspections are very similar by construction.^7^

Appendix C3 presents the distributions of the Five-Star Staffing Composite with the instability measure weighted by 10–100 points by nursing home characteristics. Increasing the weight, as expected, increases the impact of this measure substantially, with approximately 40% of nursing homes experiencing either a gain or a loss of stars.

## DISCUSSION

In this paper we argue that the Five-Star Staffing Composite should be expanded to include a staffing instability measure. The rationale supporting this argument is based on 2 facts. First, this measure has been shown in prior studies to capture an important structural aspect of the staffing quality domain in nursing homes that is associated with resident health outcomes. Second, this measure offers additional information about quality over and above the currently included measures, average staffing levels, and turnover. In fact, leaving instability out of the Five-Star Composite likely results in an incomplete and potentially biased perception of staffing quality. This can potentially mislead consumers and their agents (Medicare, Medicaid, other payers) when choosing nursing homes, ultimately frustrating CMS’ objectives to improve the market through better information about quality, to lead consumers to make more rational choices, and to enhance incentives for providers to improve quality.

The paper provides an analysis of the impact of including instability in the Five-Star Staffing Composite for 3 cases, with varying weights assigned to the measure. Not surprisingly, it demonstrates that the number of nursing homes losing a star or gaining a star when instability is added depends on the weight assigned to the instability measure—the higher the weight, the larger the deviation from the current distribution of the Five-Star Staffing Composite and the larger the number of losers and gainers.

These analyses raise the question of what the appropriate weight for the instability measure might be. The choice reflects the value that the decision maker assigns to instability relative to the other measures included in the Five-Star Staffing Composite. In general, several criteria can be considered. Arguably, the most important criterion is the magnitude of the association of each staffing measure with resident outcomes. Prior research shows, as we discuss above, that all 3 staffing measures are associated with outcomes, although not to the same degree for a given outcome or across different facilities, leading to different ranking for the same facilities and the need to include all 3. This makes the choice based on relationship to outcomes value-based, depending on which outcomes one values most. It also might lead one to argue in favor of a person-chosen, individualized Five-Star measure, rather than, or as another option to, the CMS constructed composite.^28,29^ Furthermore, a recent study^16^ identified methodological limitations with much of the evidence base regarding the average staffing/outcome relationships, suggesting the measures’ accuracy should be another important criterion. Experience with a measure and practitioners being accustomed to using and cognitively comfortable with it might increase its value as well. And finally, if a new measure is proposed with which practitioners do not have much experience, a lower weight might be appropriate to minimize risk of the change, while still allowing the measure to have influence when it reflects particularly good or poor outcomes.

For our base case we chose a middle-of-the-road weight for instability of 5–50, equal to the weight used for several other recently added measures. We also performed alternative analyses with weights of 10–30 and 10–100, the 2 extreme cases that CMS uses for other staffing components. To assess the “riskiness” of each measure, we compared agreement on nursing homes’ assignments with the current Five-Star Staffing Composite when instability was added. The base case seems conservative, with only approximately 20% of nursing homes experiencing a change in the staffing and the Overall Five-Star. The case with the 10- to 100-point weight, a weight commensurate with weight given to average staffing, impacts approximately 40% of nursing homes. Our judgment is that the base case is the most appropriate, given the tradeoffs between capturing its potential impact on resident outcomes while minimizing disruption due to a relatively new measure. Although opinions may vary, the recent National Academies of Sciences, Engineering and Medicine (NASEM) report recommended that all staffing measures be given more weight,^30^ particularly since the PBJ data became available.^12,31,32^ Ultimately, this is a policy decision to be determined by the values and trade-offs of policymakers and other stakeholders.

## Conclusion

Any public reporting system should be dynamic, with included measures and scoring methodology adapting to newly available data, measures, and standards, such as the average staffing standards proposed by CMS.^33^ The CMS has been adapting its reported nursing home quality measures over the last 2 decades to reflect these factors. In recent years, research has made clear that staffing instability—the daily fluctuations in the adequacy of staff that may be masked when using average staffing levels, whether based on actual averages or newly promulgated standards—is an important aspect of quality that consumers should be made aware of, and providers should seek to improve. Adding a measure of staffing instability to NHCC would meet these goals.

## Supplementary Material

qxae159_Supplementary_Data
