# Supplementary material for: Incorporating staffing instability in the nursing home Five-Star Staffing Composite
Source: Health Aff Sch. 2024 Dec 16;2(12):qxae159. doi: 10.1093/haschl/qxae159 (PMC11646700; doi:10.1093/haschl/qxae159)

Appendix A: Additional information about the CMS methodology of calculating the 5-Star Staffing Composite

Following CMS, we included the job codes 5 through12 in the PBJ: RN director of nursing, RN with and without administrative duties, LPNs with and without administrative duties, CNAs, aides in training, and medication aides/technicians. Excluded from the calculation were days in which resident census was zero.

We followed the CMS methodology when one of the component measures for the staffing measures is missing is to base the rating on the available measures and rescale the total. Following this methodology for nursing homes missing instability or any other measure we used the following formula to rescale their score: total points for available measures X (430/maximum possible points for available measures).

Appendix B: Impact of Including Instability in the Nursing Home Care Compare 5-Star Staffing Composite: Alternative Weights

Table 1	**5-Star Staffing Composite Rating with Instability<br>Maximum points possible = 30**					
**Current CMS 5-Star Composite Staffing Rating**	1	2	3	4	5	Total
1	2,366 (88%)	312 (12%)	0	0	0	2,678
2	98 (3%)	2,658 (85%)	389 (12%)	0	0	3,145
3	0	200 (6%)	2,732 (84%)	324 (10%)	0	3,256
4	0	0	226 (8%)	2,638 (88%)	149 (5%)	3,013
5	0	0	0	154 (10%)	1,395 (90%)	1,549
Total	2,464	3,170	3,347	3,116	1,544	13,641

Table 2	**5-Star Staffing Composite Rating with Instability<br>Maximum points possible = 100**					
**Current CMS 5-Star Composite Staffing Rating**	1	2	3	4	5	Total
1	1,948 (73%)	716 (27%)	14 (1%)	0	0	2,678
2	491 (16%)	1,691 (54%)	955 (30%)	0	0	3,145
3	4 (0%)	767 (24%)	1,644 (50%)	841 (26%)	0	3,256
4	0	12 (0%)	764 (25%)	1,780 (59%)	457 (15%)	3,013
5	0	0	1 (0%)	564 (36%)	984 (64%)	1,549
Total	2,443	3,186	3,378	3,193	1,441	13,641

Appendix C1: Impact of including Instability in the Nursing Home Care Compare Composite on Overall 5 Star - Simulation Results

Base case: Instability Measure weighted at 5-50 Points

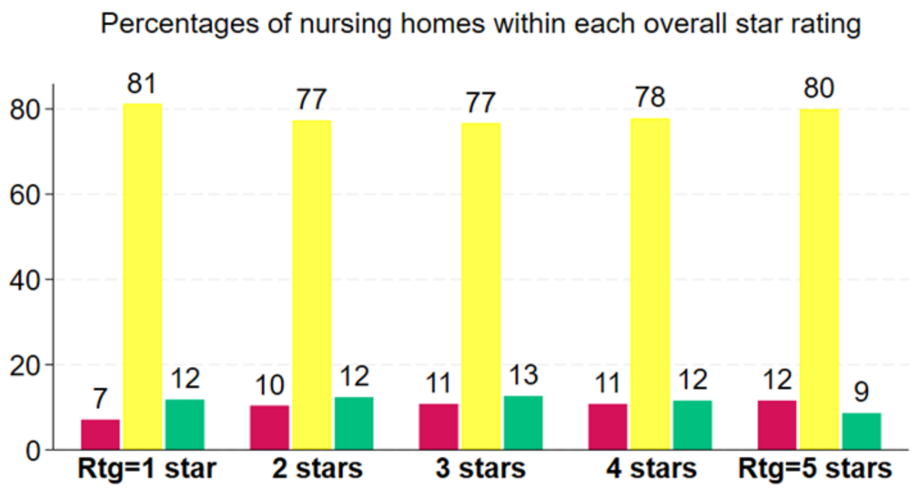

Appendix C2: Impact of including Instability in the Nursing Home Care Compare Composite on Overall 5 Star - Simulation Results

Base case: Instability Measure weighted at 10-100 Points

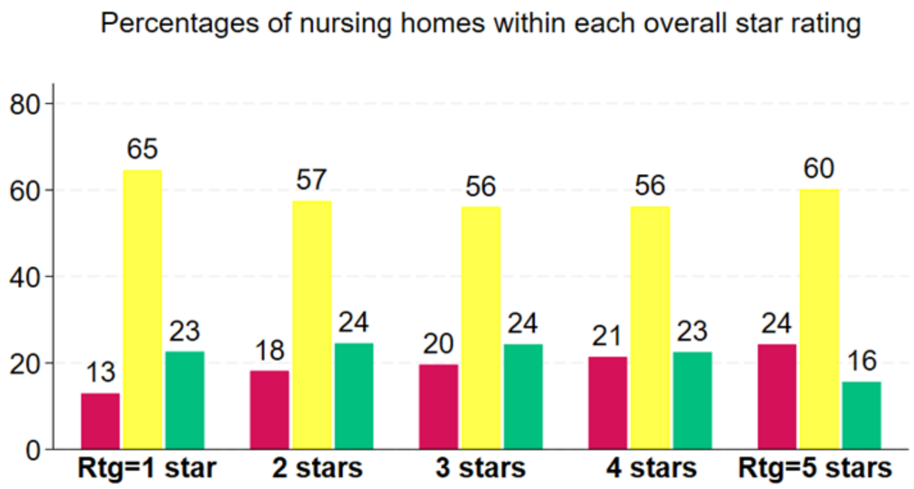

Appendix C3: Impact of including Instability in the Nursing Home Care Compare Composite By nursing Home Characteristics – Simulation Results. Sensitivity Analysis: Instability Measure weighted at 10 – 100 Points

Percentages of nursing homes within each bed size category

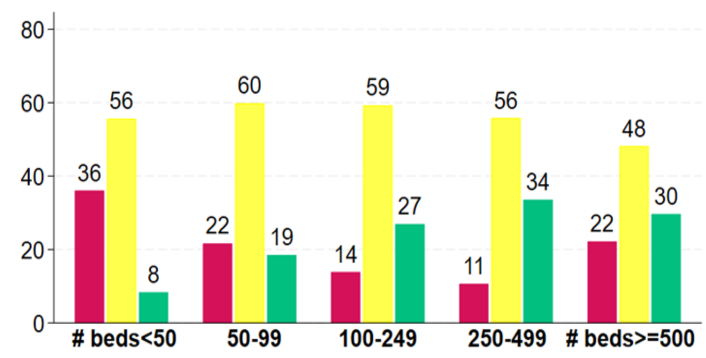

Percentages of nursing homes within each census region

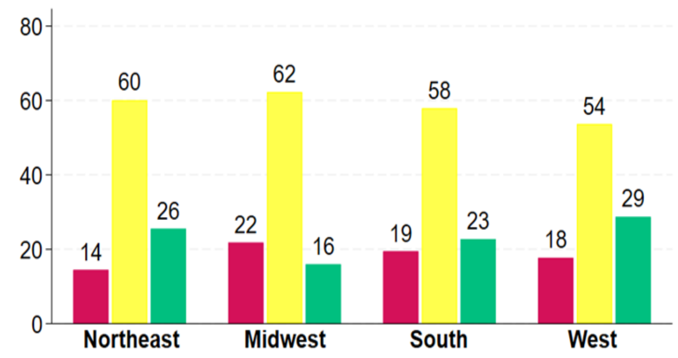

Percentages of nursing homes within each organization type

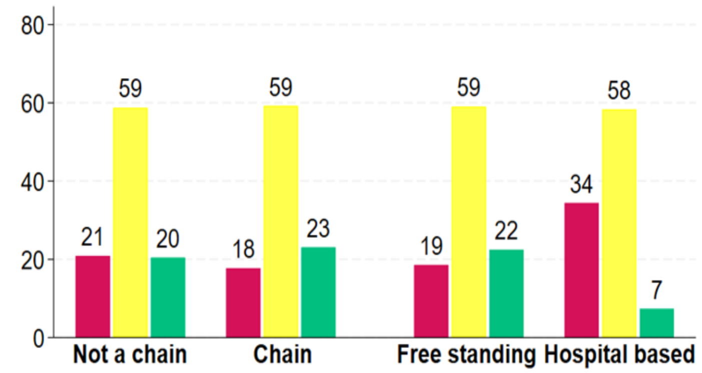

Percentages of nursing homes within each ownership type

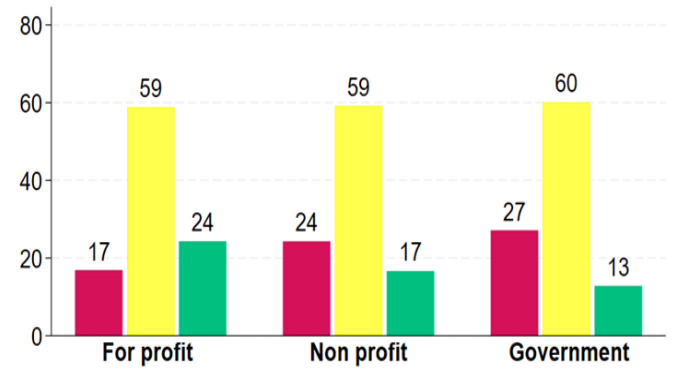

Percentages of nursing homes within each quality measures star rating

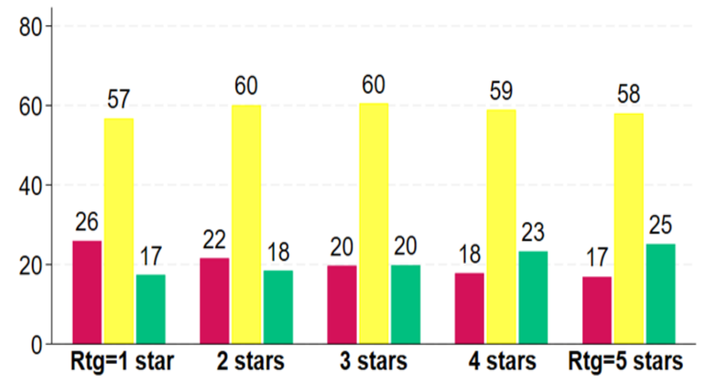

Percentages of nursing homes within each health inspections star rating

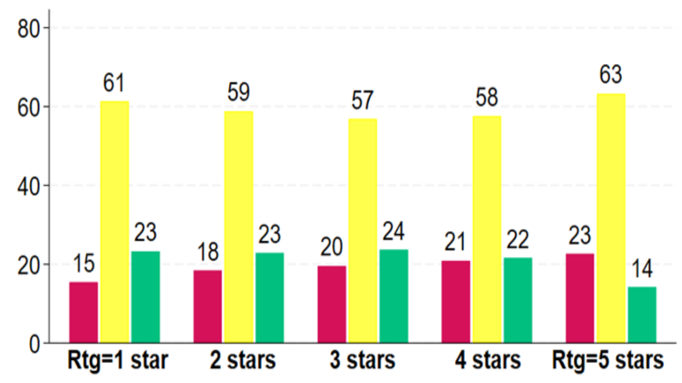

Supplement: qxae159_Supplementary_Data [file qxae159_supplementary_data.zip › Appendices a, b, c.pdf]
